# Cognitive, emotional, and social factors promoting psychosocial adaptation: a study of latent profiles in people living in socially vulnerable contexts

**DOI:** 10.3389/fpsyg.2024.1321242

**Published:** 2024-04-12

**Authors:** Nuria Carriedo, Odir A. Rodríguez-Villagra, Sebastián Moguilner, Juan Pablo Morales-Sepulveda, Daniela Huepe-Artigas, Vicente Soto, Daniel Franco-O’Byrne, Agustín Ibáñez, Tristan A. Bekinschtein, David Huepe

**Affiliations:** ^1^Departamento de Psicología Evolutiva y de la Educación, National Distance Education University (UNED), Madrid, Spain; ^2^Institute for Psychological Research, University of Costa Rica, Sabanilla, San José, Costa Rica; ^3^Neuroscience Research Center, University of Costa Rica, San José, Costa Rica; ^4^Global Brain Health Institute, Trinity College Dublin, Dublin, Ireland; ^5^Cognitive Neuroscience Center, Universidad de San Andrés, Buenos Aires, Argentina; ^6^Latin American Brain Health, Universidad Adolfo Ibáñez, Santiago, Chile; ^7^University of Sydney Business School, Darlington, NSW, Australia; ^8^Facultad de Educación Psicología y Familia, Universidad Finis Terrae, Santiago, Chile; ^9^Center for Social and Cognitive Neuroscience, School of Psychology, Universidad Adolfo Ibáñez, Santiago, Chile; ^10^National Scientific and Technical Research Council (CONICET), Buenos Aires, Argentina; ^11^Cambridge Consciousness and Cognition Lab, Department of Psychology, University of Cambridge, Cambridge, United Kingdom

**Keywords:** psychosocial adaptation, latent profiles analyses, socially vulnerable populations, social adaptation, affective, cognitive and social factors

## Abstract

**Introduction:**

Social adaptation is a multifaceted process that encompasses cognitive, social, and affective factors. Previous research often focused on isolated variables, overlooking their interactions, especially in challenging environments. Our study addresses this by investigating how cognitive (working memory, verbal intelligence, self-regulation), social (affective empathy, family networks, loneliness), and psychological (locus of control, self-esteem, perceived stress) factors interact to influence social adaptation.

**Methods:**

We analyzed data from 254 adults (55% female) aged 18 to 46 in economically vulnerable households in Santiago, Chile. We used Latent profile analysis (LPA) and machine learning to uncover distinct patters of socioadaptive features and identify the most discriminating features.

**Results:**

LPA showed two distinct psychosocial adaptation profiles: one characterized by effective psychosocial adaptation and another by poor psychosocial adaptation. The adaptive profile featured individuals with strong emotional, cognitive, and behavioral self-regulation, an internal locus of control, high self-esteem, lower stress levels, reduced affective empathy, robust family support, and decreased loneliness. Conversely, the poorly adapted profile exhibited the opposite traits. Machine learning pinpointed six key differentiating factors in various adaptation pathways within the same vulnerable context: high self-esteem, cognitive and behavioral self-regulation, low stress levels, higher education, and increased social support.

**Discussion:**

This research carries significant policy implications, highlighting the need to reinforce protective factors and psychological resources, such as self-esteem, self-regulation, and education, to foster effective adaptation in adversity. Additionally, we identified critical risk factors impacting social adaptation in vulnerable populations, advancing our understanding of this intricate phenomenon.

## 1 Introduction

Living in disadvantaged socioeconomic environments requires confronting multiple kinds of adversities, beyond an absolute or relative lack of resources. The existing threats in these environments become frequent at both the community and individual levels. At the larger social level, impoverished environments are associated with higher crime rates, drug dealing, substance abuse, disease rates, and limited access to social security, education, housing, or jobs (Chaudhuri, 2003; Evans et al., 2005). The aggregate effect of these threats creates a spiral of toxic stress that leads individuals to maladaptation, ultimately overwhelming the person’s ability to cope and hindering cognitive function in adults (Buckner and Waters, 2011; Neely-Prado et al., 2019).

There is growing evidence suggesting that individuals residing in chronically stressful environments or exposed to highly disruptive and threatening events exhibit stable yet distinct trajectories of adaptation (Galatzer-Levy et al., 2018; Tao et al., 2023). According to one of the predominant theories in the field, the Conservation of Resource (COR) Theory (Hobfoll, 1989; Hobfoll, 2002; Hobfoll et al., 2015; Holmgreen et al., 2017), these trajectories are influenced by individuals’ possession and accessibility of various resources and their efforts to prevent resource depletion. Furthermore, resources tend to coalesce rather than occur independently, forming dynamic resource caravans that can be established or dissolved over time, thus generating gain/loss spirals or cascades (Tao et al., 2023). This viewpoint is consistent with other theories on resources and resilience, underscoring the significance of personal and social resources in navigating adversity and chronic stress (e.g., Lazarus and Folkman, 1984; Baltes, 1997; Holahan et al., 1999; Bonanno and Diminich, 2013). Importantly, the Selection, Optimization, and Compensation (SOC) theory (Baltes, 1997) further proposes that individuals strategically select, optimize, and compensate for available resources to address cumulative and episodic processes of gain and loss throughout the lifespan.

Therefore, notwithstanding challenging circumstances that may precipitate resource loss, both COR and SOC theories posit that effectively addressing these challenges can result in augmented resources. Considering the variability of interacting resources and the accompanying theories, attempting to succinctly summarize the mechanisms by which they foster social adaptation and well-being appears impractical (Hobfoll, 2002). However, as Hobfoll has emphasized, there are common elements shared among diverse resources: firstly, individuals are motivated to create and safeguard resources. Secondly, those endowed with resources are less susceptible to the adverse effects of stressful circumstances. Thirdly, individuals with a variety of available resources are better equipped to selectively confront stressful situations. Fourthly, individuals with more resources are less adversely affected by resource depletion during stress and can replenish or absorb losses by tapping into additional reserves. Fifthly, resources are interconnected, fostering enrichment among those with substantial resource reservoirs. Sixthly, the influence of resources is long-term and tends not to be transient across time and different circumstances. Seventhly, within a particular culture, resources are sought after because they contribute to success, resilience against stress, and are inherently valued.

Additionally, Masten et al. (2021) have proposed a dynamic interplay of multiple and complex adaptive systems (person, family, community) where diverse resources interact, highlighting that certain resources may exhibit promotive effects (main effect) or protective effects (variable effect depending on the level of risk) against maladaptation. However, interaction effects observed in variable-centered studies may indicate divergent processes among individuals, implying that a particular variable or resource that is protective for some individuals could exert negative effects on others, and that these effects could vary across the individual’s development, circumstances and contexts.

Consequently, while to our knowledge, COR theory has not been directly applied to elucidate social adaptation in socioeconomically impoverished contexts, it posits that not all individuals in such environments encounter adverse outcomes uniformly. This is because the interaction of diverse factors could engender distinct pathways of adaptation/maladaptation (Bonanno and Diminich, 2013). Therefore, when confronted with adverse stressful environments, some individuals may adeptly navigate challenges and undergo satisfactory socioemotional adjustment, while others may endure varying levels of distress, resulting in detrimental consequences for social adaptation and well-being (also see Masten et al., 2021).

Based on this premise, we hypothesize that it is plausible to anticipate distinct latent profiles among individuals pertaining to the interplay of cognitive, emotional, and social variables, particularly among those residing in vulnerable contexts. Furthermore, we propose that these profiles may variably predict psychosocial adaptation. Consequently, the objective of the current study was to delineate these latent profiles, discern significant differentiators among them, and assess their respective capacities to predict varying levels of individuals’ psychosocial adaptation at a given juncture.

### 1.1 Social adaptation and psychological-well-being as indicators of psychosocial adaptation in economically vulnerable populations

Social adaptation (SA) can be considered the capacity to confront, relate to, compromise, and cooperate with others. SA involves accommodating thoughts and behaviors, emotional regulation, promotes rewarding social interactions and facilitates social sensitivity (Ma et al., 2016). Moreover, SA also considers effective coping skills, healthy interpersonal relationships, and an individuals’ motivation to participate in their various societal roles while interacting with others according to social norms and expectations (López et al., 2016; Samadi and Sohrabi, 2016). Conversely, maladaptive behavior imposes stress on social systems; primarily because it breaks established norms and conventions for socialization (Liu et al., 2021). Maladaptive behaviors are more frequently observed in vulnerable contexts where people with insufficient income and low educational levels are more likely to live (Thompson and Dahling, 2019; Papadakis et al., 2020), also affecting overall feeling of psychological well-being (PWB).

SA and PWB as such, are moderately related constructs linked to social contacts, relationships, friendship, and adaptive social functioning (Lent, 2004; Blanco and Díaz, 2005; Masten, 2014) which reflect individual levels of adjustment (Keyes, 2002). Both require individuals to associate, accommodate, compromise, cooperate and cope with the environment and others (Samadi and Sohrabi, 2016).

According to Lent (2004), positive adjustment is indeed a multifaceted concept that incorporates cognitive, affective, and behavioral dimensions. It transcends mere satisfaction or contentment and encompasses achieving “satisfactoriness” which involves meeting environmental demands and expectations. This includes fulfilling responsibilities across different life domains and roles. Thus, a comprehensive view of positive adjustment should recognize both intrapersonal and interpersonal functioning. It should consider subjective feelings of well-being alongside objective assessments of effectiveness and fulfillment in life. This holistic perspective enables a deeper understanding of individuals’ adaptive capacities and overall adjustment in various contexts.

In accordance with this perspective, we can operationalize psychosocial adaptation as the by-product of SA and PWB. Thus, psychosocial adaptation includes individual and social dimensions of adult functioning (Keyes, 1998) reflecting the subjective experience of quality of life, sense of control, and self-esteem (Huepe et al., 2011).

Taken together, previous research highlighted that not all individuals living in impoverishing contexts show maladaptation (Bonanno and Diminich, 2013) because the interaction of cognitive, emotional, and social factors could lead to different pathways of psychosocial adaptation/maladaptation (Masten et al., 2021). However, this previous research is mainly focused on the study of single-variable outcomes, with few on the interrelations between them. Some systematic reviews noted that few studies included more than six variables, arguing the necessity of controlling the interaction among them and for more complex models that simultaneously consider multiple variables (Fritz et al., 2018b). To fill this gap, we aimed to find different profiles of psychosocial adaptation through latent profile analysis (LPA), a person-centered approach that allows to find different latent profiles within the same group of people based on the interindividual differences in multiple variables that are considered simultaneously in the analysis.

### 1.2 The present study

Here, we aim to identify and validate different profiles of social, emotional, and cognitive characteristics related to psychosocial adaptation among adults living in impoverished environments. To address this question, we used a combined methodological approximation: (1) a theoretically driven LPA to identify distinct profiles and (2) data-driven machine-learning methods to validate group classification. Furthermore, we explored whether specific latent profiles are associated with psychosocial adaptation (second research question) and eventually determined the most critical variables that account for this classification through a progressive feature elimination machine learning approach (third research question).

The selection of the features to introduce as indicators to the LPA was carried out through a literature review on psychosocial adaptation and related fields, such as resilience or well-being, both in adults and across development. Most of the revised studies and reviews came from developmental science. Across them, the resources identified have been described as protective factors that are generalizable and operate similarly in different populations across the lifespan (Holahan et al., 1999).

Although studies on the adult population are scarce, some have also identified socio-affective and cognitive resources or resilience factors that could predict psychosocial adaptation (Hobfoll, 2002; Huepe et al., 2011; Huepe and Salas, 2013a; Neely-Prado et al., 2019; Franco-O’Byrne et al., 2023), but few of them have considered multiple variables simultaneously (Fritz et al., 2018b).

This is a critical issue because the different variables or resources act orchestrated (Hobfoll, 1989; Holahan et al., 1999; Hobfoll, 2002; Hobfoll et al., 2015; Holmgreen et al., 2017), tend to causally generate other resources (Holahan et al., 1999), or have compensatory or protective effects in the context of adversity (Baltes, 1997; Hobfoll, 2002; Masten et al., 2021). Moreover, as people may use an array of resources or resilience-promoting factors to cope with adversity (Bonanno and Diminich, 2013) it is critical to assess the most relevant variables simultaneously, to find different profiles of psychosocial adaptation.

Taking these considerations into account and based on the evidence from different single studies, meta-analyses, and systematic reviews on the topic (Meng et al., 2018; Fritz et al., 2018b; Gartland et al., 2019a; Yule et al., 2019a), we selected the variables to be included in this study. All the chosen variables were significant when tested in isolation, but some lost significance simultaneously because of their cumulative or suppressing effects (Bonanno et al., 2011a,b). Also, some of these variables are modified or mediated by other factors (Bonanno and Diminich, 2013; Fritz et al., 2018b); thus, their effects may not be fully independent (Hobfoll, 2002).

### 1.3 Variable selection for the LPA

Across the scientific literature there is a strong consensus regarding the importance of self-regulation for SA (i.e., impulse control, emotional regulation, metacognition); self-esteem; social support; locus of control; empathy; and cognitive factors as intelligence or executive functioning (Buckner et al., 2003; Buckner and Waters, 2011; Meng et al., 2018; Gartland et al., 2019b), although it has been shown that the relative importance of these factors could vary across cultures (Masten et al., 2021).

*Self-regulation* must be considered an essential factor for SA. It modulates and monitors thoughts, emotions, and behaviors (Flouri et al., 2014a). It allows both to cope with stressors before they occur (anticipation) and after they have occurred, reducing their impact by managing negative emotions and thoughts (Aspinwall and Taylor, 1997; Buckner et al., 2009). It has been well established that self-regulatory abilities promote good adaptive functioning in diverse areas such as social relationships, self-reflection, mental health, or social competence (Buckner et al., 2009). Some aspects of self-regulation encompass abilities regarding emotion regulation, executive functions, or impulse control. A recent systematic review by Fritz et al. (2018b) found that factors related to self-regulation such as cognitive reappraisal, low rumination, high distress tolerance, and low expressive emotion suppression are resilience factors. Therefore, we expect high cognitive, emotional, impulse control, and self-regulation to positively affect psychosocial adaptation. As such, for indicators of self-regulation we have selected control of thoughts, emotional regulation, and impulse control.

There is abundant empirical evidence supporting the role of *self-esteem* as a resilience factor and resource for SA in adults and children. Self-esteem is negatively related to social stress (Kesting et al., 2013) and higher symptoms of anxiety and depression (Dumont and Provost, 1999; Sowislo and Orth, 2013) but positively associated with higher subjective well-being (Orth and Robins, 2014; Pu et al., 2017) and psychosocial adaptation in adults living in vulnerable contexts (Neely-Prado et al., 2019). In line with these results, we expect high self-esteem will be essential for adequate psychosocial adaptation in these populations.

Furthermore, prior studies have also underlined the role of *social support* in promoting SA (Franco-O’Byrne et al., 2023). Social support comprises family resources, supportive interactions, subjective perceptions of support, or the presence of intimate others (Hobfoll, 2002). People with more social support experience higher PWB and are more resistant to stress (Dumont and Provost, 1999; Anthony, 2008; Gartland et al., 2019) identified 11 studies that found social support outside the family (friends or adults) as a significant factor in the face of poverty. Therefore, we expect that high social support from family and friends will contribute positively to psychosocial adaptation. As for indicators of social support, we have used the feeling of loneliness and family networks.

*Internal locus of control* must be also considered as a factor enhancing SA. It alludes to people’s perception of whether life outcomes depend on personal inner factors instead of external ones. Internal locus of control has been proposed as one of the most influential personality characteristics in enhancing psychological and social well-being and as a resource for coping with stress (Borman and Overman, 2004; Nguyen and Joel Wong, 2013; Meng et al., 2018; Fritz et al., 2018b; Gartland et al., 2019; Neely-Prado et al., 2019). The Kauai Longitudinal Study (Werner, 1996, 2015), which followed 698 poor children until adulthood, found that resilient people have a more internal locus of control and high self-esteem. Therefore, we predict that a high internal locus of control will promote psychosocial adaptation.

Existing studies have previously addressed the relation of *empathy* to SA (Neely-Prado et al., 2019). Empathy has been considered a motivator for prosocial behavior (Decety et al., 2016; Tomova et al., 2017) and social behavior (Wolf et al., 2015), and it may be a natural inhibitor of violent behaviors (Smith, 2006). Furthermore, affective sharing with others and self-regulatory abilities that allow self-monitoring and reflection, critical factors for sustaining productive and supportive interactions with family, friends, and community (Eslinger and Long, 2016). Moreover, during empathic processes, contextual cues activate previous experiences that allow us to predict others’ intentions, feelings, and behaviors, allowing the coordination of previous social, emotional, and cognitive experiences (Melloni et al., 2014). This contextual modulation could promote flexible adaptation to different social contexts. However, some other studies also found that early life stress is associated with decreased emotional empathy and increased vulnerability to depression (Grimm et al., 2017; Cameron et al., 2019). Therefore, although previous research led us to predict that high affective empathy would promote psychosocial adaptation, it may not be the case for all individuals living in contexts of chronic stress (Eisenberg and Eggum, 2009). That is, in some stressful situations, emotional feelings of sorrow or concern for others could lead to personal distress, which may depend on the individual’s abilities to regulate their emotions. For indicators of empathy, we have chosen affective empathy conceptualized as empathic concern and personal distress.

Similarly, *stress* resistance has also been identified as a critical factor in adjustment to adversity (Masten et al., 2021). Poor people have fewer material resources to cope with stressful situations and are frequently exposed to a considerable variation of stressors (Buckner and Waters, 2011) and, in turn have fewer opportunities to experience positive stressors, that is, situations that pose challenges that drive people to success or growth. Chronic exposure to stress in some contexts as poverty, could have a negative impact on cognitive and socio-affective domains (Neely-Prado et al., 2019). However, individual differences (developmental stage, personality traits, coping strategies, lifestyle, trauma/abuse history, social support, among other factors) determine sensitivity to chronic stress and are crucial to understanding different pathways of resiliency or maladaptation (Yuen et al., 2009; McEwen, 2012; Cicchetti, 2013; De Minzi and Lemos, 2014; Meng et al., 2018). The Kauai longitudinal study (Werner, 1996, 2015) found that resilient adolescents showed more flexibility in dealing with stress, high cognitive abilities, good impulse control, a reflective cognitive style, more internal locus of control, and higher self-esteem. Similarly (Aspinwall and Taylor, 1997) pointed out that self-regulation skills are essential to cope with stress by anticipating, preventing, and acting on stressors while they occur. Therefore, we predict that low perceived stress will contribute to better psychosocial adaptation.

*Cognitive abilities* have also been related to SA in the literature. Under the umbrella of cognitive abilities, very different variables that allow the capacity to reason and solve problems are included. Based on previous results, we selected two indicators of cognitive abilities: working memory and verbal intelligence. *Working memory* is an important cognitive function that has been related to cognitive control and executive functioning (Miyake et al., 2000), intelligence (Ackerman et al., 2005; Kane et al., 2005), and self-regulation (Bogg and Finn, 2010; Baird et al., 2013). It is essential for reading comprehension, reasoning, and problem-solving in children and adults (Carriedo et al., 2011; Iglesias-Sarmiento et al., 2015, 2023), and are abilities necessary for academic achievement, and professional success. Poverty in childhood has been linked to low working memory in adulthood, although chronic stress mediated its effect (Evans and Schamberg, 2009). Moreover, working memory has been proposed as important for promoting adaptive factors in adults who live in vulnerable contexts (Levens et al., 2017; Neely-Prado et al., 2019), and it has also been related to resilience (Curtis and Cicchetti, 2003; Bemath et al., 2020). In turn, *verbal intelligence* has been proposed to be another important factor in facing adversity (Neely-Prado et al., 2019). Specifically, poor children with high verbal cognitive ability may be more successful in assessing and obtaining resources from their environment and handling adversity-related problems (Masten et al., 1999). However, evidence regarding verbal intelligence is inconsistent. Some other studies (e.g., Brown et al., 2013) have found only moderate support for this association in a large longitudinal cohort of 1,603 boys living in poverty.

In light of the identified predictors’ impact on psychosocial adaptation, we anticipate that the interplay among social, cognitive, and affective resources (including social support, stress, cognitive abilities, self-esteem, empathy, self-regulation, and locus of control) will enable the differentiation of various constellations of variables (profiles) that characterize various groups of individuals. These profiles are likely to delineate distinct trajectories of adjustment (functionality) within socially vulnerable contexts. Drawing upon prior research (Holmgreen et al., 2017; Masten et al., 2021), alongside an anticipated pattern of poor psychosocial adaptation, we expect to observe at least one pattern indicative of favorable psychosocial adaptation. In this pattern, individuals are expected to demonstrate high levels of self-regulation and self-esteem, an internal locus of control, substantial social support from both family and friends, low levels of perceived stress, enhanced cognitive abilities, and heightened affective empathy.

Additionally, as discussed above, *psychosocial adaptation* was operationalized as the by-product of SA and PWB. However, while they are related (Lent, 2004; Blanco and Díaz, 2005; Masten et al., 2021), they are not identical. Based on their communality (see Supplementary Figure S1 for a Confirmatory Factor Analysis), we hypothesized that the expected profiles will be associated with the indicators of SA and PWB in the same direction.

## 2 Methods

### 2.1 Participants

Two hundred sixty-one participants were recruited to take part in the study (female: 146; age: *M* = 31.7, SD = 7.9, range = 18–46; years of education: *M* = 13.6, SD = 3.03. male: 115; age: *M* = 31.5, SD = 8.03, range = 18–46; years of education: *M* = 13.8, SD = 2.67). After removing outliers (see specific details in the Results section) a total of 254 adults (142 female, 55%) between ages 18 to 46 (*M* = 31.63, SD = 7.95) remained. In regards to formal education, participants reported between 5 to 22 years (*M* = 13.68, SD = 2.87); this mean is equivalent to having completed high school. Participants were recruited by accessibility. All participants were adults from Santiago de Chile, and belonged to the 40th percentile of households with the lowest income and more vulnerability, as recorded by the Chilean Welfare Program (Ministry of Social Development and Family, 2020). Only those who had the Social Registry of households^1^ were recruited. People or families in this condition correspond to a low socioeconomic status because within this percentile, they show marked disadvantages in various socioeconomic indexes, including household income, level of disability/dependence, possession of goods, and access to public services. We excluded individuals with a visual or hearing impairment who told us they would be unable to complete our assessment battery (e.g., to read or respond to verbal information or to follow the evaluator’s oral instruction). Any psychiatric or psychological disorders from participants were ruled out through a previous interview.

### 2.2 Procedure

Trained professionals from social sciences applied all sociodemographic, neuropsychological tests, and psychological questionnaires used in this study. The tasks were presented on a computer screen with the assistance of a research assistant; the participants were asked to sit comfortably and respond to the set of scales and questionnaires. Participation was voluntary and all participant data was anonymized using an alpha-numeric coding procedure. Evaluators made sure that each participant understood the informed consent before starting the application of the tests. The participants could inquire to the examiner in case of any doubt. The order of the tests was random to avoid any bias. The participants took approximately 3 h to complete the protocol, with a 15-min break in the middle. The field study was carried out between 2017 and 18, prior to the sars-cov-2 (COVID-19) pandemic.

All procedures were performed in accordance with the 1964 Helsinki Declaration and its later amendments or comparable ethical standards. The Universidad Adolfo Ibáñez ethics committee approved every procedure for this project. In accordance with this, all participants were provided information sheets and signed informed consent forms prior to testing.

### 2.3 Materials

The protocol included measures of self-regulation, self-esteem, social support, locus of control, affective empathy, perceived stress, working memory, and verbal intelligence. In Supplementary materials (Section A) can be seen a complete description of the instruments as well as their estimates of reliability. All the reliability estimates for this sample were adequate, ranging between *α* =0.60 and *α* = 0.91. We briefly describe the instruments used here.

Self-regulation was evaluated by three tests devoted to measuring cognitive, emotional, and behavioral components. The assessment of cognitive components was performed using the Metacognition Questionnaire 30 (MCQ-30) subscale of Control of Thoughts (Wells and Cartwright-Hatton, 2004). Self-regulation of emotions was measured with the subscale of Cognitive Re-evaluation of the Emotional Regulation Questionnaire (ERQ). Behavioral self-regulation was assessed with The Barrat Impulsivity Scale (BIS-11) (Patton et al., 1995). Self-esteem was measured using the Rosenberg Self-esteem Scale (Rosenberg, 1965). Social support is measured through two tests: the UCLA Loneliness Scale (Russell, 1996) and the subscale of Family Network of Lubben Social Network Scale–Revised (LSNS-R) (Lubben, 1988). Internal locus of control was assessed through the subscale of Internality of the Attributional Style Test (Levenson, 1974). Affective empathy was measured using the Empathic Concern and Personal Discomfort subscales of the interpersonal Reactivity Index IRI (Davis, 1983). Perceived stress was evaluated through the Perceived Stress Scale, PSS (Cohen et al., 1983). We also measured two cognitive abilities through two subscales of the WAIS-IV (Wechsler, 2003); the WAIS-IV – Digit Span Backwards for working memory and the WAIS-IV – Vocabulary as a measure of verbal intelligence. As for indicators of psychosocial adaptation, we employed the Social Adaptation Self-Regulation Scale (SASS) (Bosc et al., 1997) and the Psychological Well-Being Scale (PWB) (Ryff and Singer, 2006). SASS, examines the quality of non-family relationships, work and leisure, sociocultural interests’, and family relationships. PWB, as proposed by Ryff and Singer (2006), is a broader construct that involves dimensions such as positive relationships, self-acceptance, autonomy, environmental mastery, personal growth, and purpose in life.

## 3 Data analysis

### 3.1 Latent profile analysis

LPA is a categorical latent variable modeling technique that allows the identification of latent profiles within a sample based on a set of observed variables. In the present study, we selected the set of relevant indicators for social adaptation depicted in previous sections. Data preparation and plotting were carried out in RStudio (R Core Team, 2020; R Studio Team, 2020) using the Tidyverse (Wickham et al., 2019), TidyLPA (Rosenberg et al., 2018), MplusAutomation (Hallquist and Wiley, 2018), naniar (Tierney et al., 2021) and janitor (Firke, 2021) packages. LPA models were carried out using Mplus 8.7 (Muthén and Muthén, 2018) using full maximum likelihood estimation (FIML). We employed 1,000 random starts and 500 replications to ensure that the model estimation converges on the global maximum of likelihood. LPA is a model testing process, fitting multiple models with different numbers of profiles. For model fit comparisons and selection of the optimal number of latent profiles we used a series of model fit indices and statistics, parsimony and theoretical interpretability. The statistical criteria used in the present study were: (a) the relative fit information criteria the Bayesian information criterion (BIC) and the Akaike’s information criterion (AIC); (b) a criterion for assessing the quality of profiles membership classification (entropy); and (c) a nested model test that compares neighboring models (e.g., k vs. k + 1 profiles), the Lo–Mendell–Rubin likelihood ratio test (LMR LR). In this study, we also examined the prediction of covariates gender, age, and years of education to attribute profile membership and to validate the retained profile solution with two theoretically relevant measures for psychosocial adaptation; a measure of social adaptation (SASS) and a measure of psychological well-being (PWB). To this aim, we implemented our LPA models following the three-step method (Muthén and Asparouhov, 2013; Asparouhov and Muthén, 2014). In Step 1, unconditional LPA models are estimated, and the selected model according to statistical criteria and theoretically interpretability is used in the next step. In Step 2, the most likely profile memberships are estimated from the posterior probabilities of the LPA. Furthermore, the profile-classification uncertainties (i.e., measurement error) in the latent profile membership are calculated and used in the next step. In Step 3, the relationships of the most likely latent profile membership with covariates or/and distal outcomes are estimated, considering the measurement error in the estimation of the latent profile membership. For more information about the criteria used see Supplementary materials, Section B.

### 3.2 Machine-learning methods

The XGBoost (Kaufmann et al., 2019) classifier was used for subject-group classification (i.e., Good - vs. Poor configuration for psychosocial adaptation). For more information about the criteria used see Supplementary materials, Section B. Next, we performed a progressive feature elimination to select the optimum set of features after stabilization (Donnelly-Kehoe et al., 2018) using a 5-fold cross-validation scheme. We started the feature stabilization process with the full set of features. We used the Gini scores to remove features with the lowest importance at each iteration and checked for the robustness of our results based on the final number of features after stabilization (Kingsford and Salzberg, 2008). Finally, we kept the N first features in the ranking, where N was the optimal number of features such that using more than N features fails to improve the classifier’s performance. Following best practices in machine learning (Poldrack et al., 2017), we employed a k-fold validation approach (k = 5) using 80% of the sample for training and validation, and 20% as an out-of-folds sample for testing.

## 4 Results

### 4.1 Latent profile analysis results

First, outliers were eliminated because they can bias the results of LPA. We eliminated scores that were ± 3 standard deviations (SDs) from the mean. After eliminating outliers, the total percentage of missing values for each indicator was less than 4.3% (Emotion regulation = 1.5%; Control of thoughts = 0.7%; Impulsivity = 0.0%; Working memory 1.9%; Verbal intelligence: 4.3%; Locus of control = 1.1%; Self-esteem = 1.5%; Perceived stress = 0.0%; Affective empathy = 1.1%; Family networks = 0.3%; Loneliness = 0.3%).

Table 1 shows the goodness-of-fit statistics for profiles one to three. The BIC supports a two-profile solution while the AIC selects a model including three profiles. Entropy values suggest that the cases could be appropriately allocated to the correct latent profile with adequate certainty in both models (two-profile model = 0.79, three-profile model = 0.82). Scrutiny of the relative sizes of the emergent groups in the three-profile solution revealed that 81 (31%), 154 (60%), and 19 (7%) persons were classified in profiles 1, 2, and 3, respectively. The two-profile solution revealed that 153 (60.2%) persons were classified in profile 1, and 101 (39.7%) persons were classified in profile 2. Lubke and Neale (2006) suggested rejecting profiles with fewer than 25 cases and that their retention should be carefully supported on parsimony and conceptual meaningfulness. Inspection of the three-profile solution suggested that, except for the family networks indicator, there were no other differences between Profile 3 and Profile 1. Thus, given the small size of Profile 3 and its scarce differentiation from Profile 1, the preference of BIC, the appropriate entropy value for the two-profile solution, and parsimony, we selected the two-profile solution as the best account for the data.

**Table 1:** Model fit of latent profile with up to three latent profiles.

Profiles	BIC	AIC	Entropy	LMR LRT	LMR LRT (*p*-value)
1	7943.339	7865.518	1		
2	7727.333	7607.064	0.786	278.267	0.000
3	7732.123	7569.406	0.825	60.74	0.261

BIC, Bayesian Information Criterion; AIC, Akaike’s Information Criterion; BLRT, bootstrap likelihood ratio test; BLRT (*p*-value), *p*-value for bootstrap likelihood ratio test.

In Supplementary materials, Section B, you can see Supplementary Table S1 with the main statistics for the two identified profiles for each variable.

**Figure 1:**
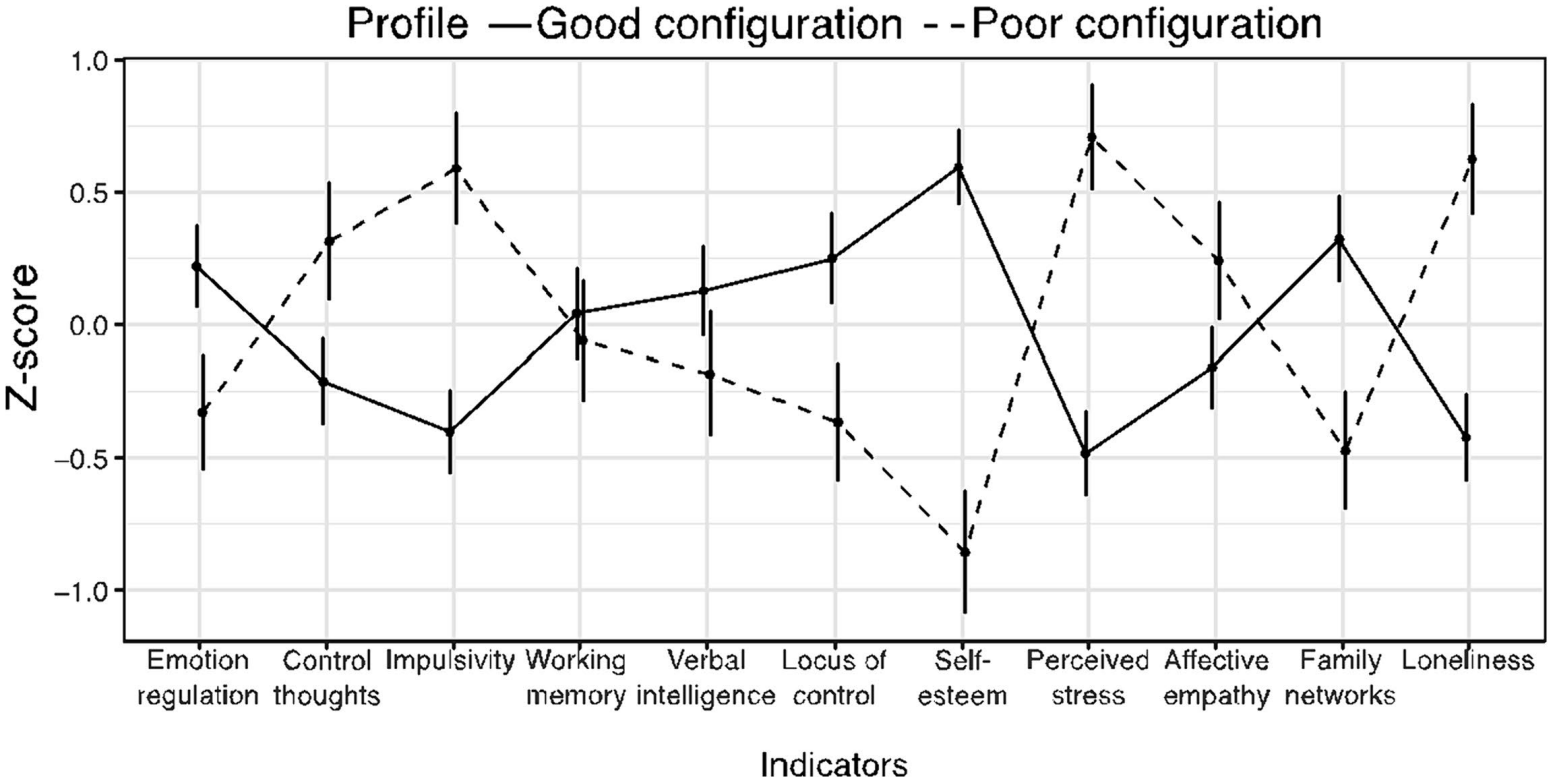
Latent class profile scores on measured variables. Bars represent 95% CIs of the mean.

Figure 1 displays the latent profiles of participants. Relative to Profile 2, individuals of Profile 1 showed significantly higher scores in emotion regulation, locus of control, self-esteem, and family networks. Furthermore, individuals in Profile 1 showed significantly lower scores in control of thoughts, impulsivity, perceived stress, affective empathy, and loneliness than those in Profile 2. Profiles did not significantly differ in working memory and verbal intelligence. Thus, the pattern of the participants’ scores in Profile 1 suggests a configuration of emotional, behavioral, and cognitive features that could facilitate psychosocial adaptation. On the contrary, the configuration of the social, emotional, and cognitive features related to subjects clustered in Profile 2 could obstruct psychosocial adaptation. Consequently, we termed Profile 1 and Profile 2 as Good and Poor configurations for psychosocial adaptation, respectively.

**Figure 2:**
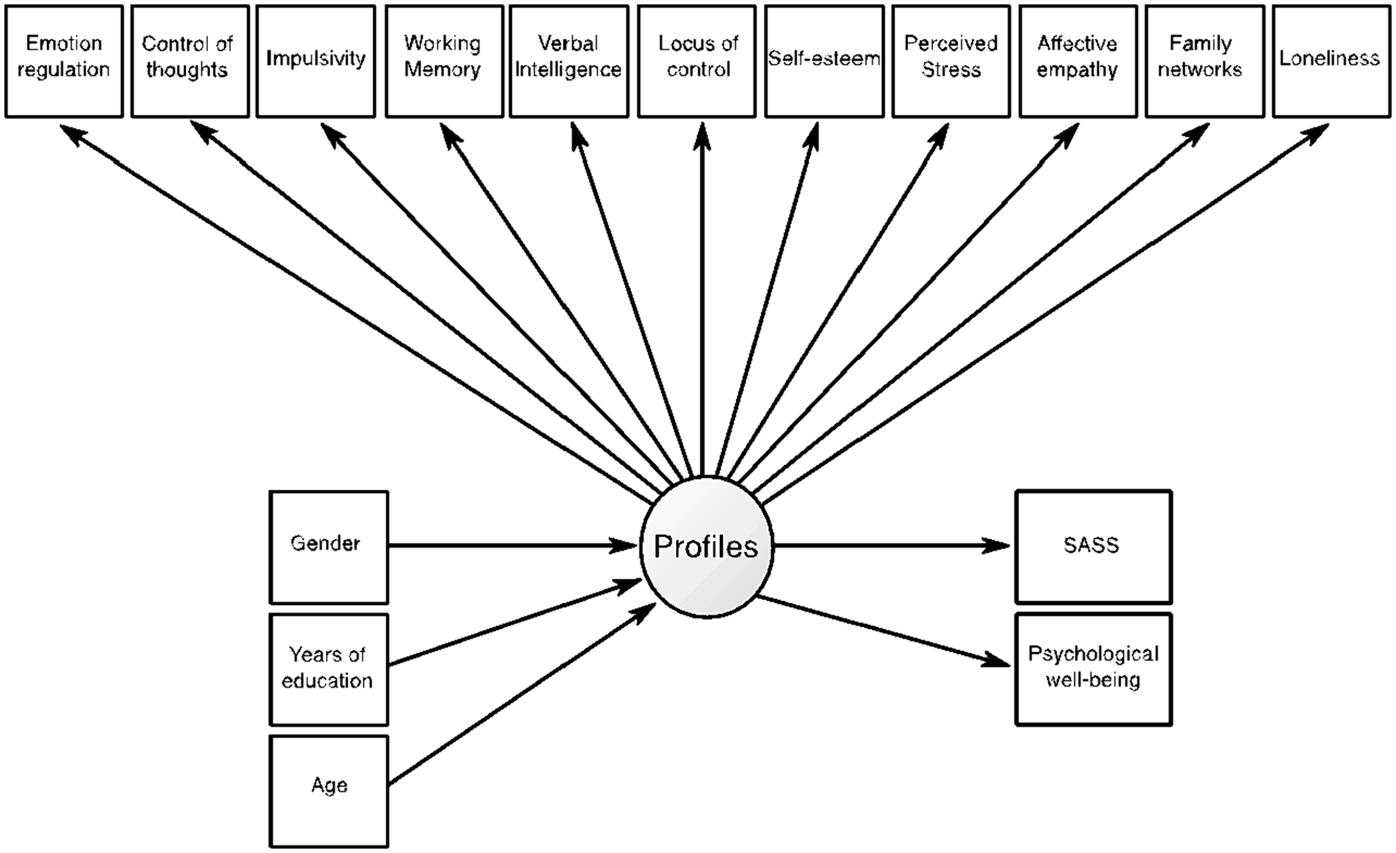
Conceptual schema of LPA implemented in Step 3. Circle: represents the latent variable with the two categorical profiles (latent variable) estimated from the selected model in Step 1; directional arrows: depict the direct effect of one variable on another; rectangles: represent measured/observed variables. In this model, the covariates (i.e., gender, years of education and age) predict the latent profiles and the latter latent variable predict the measured variables SASS and Psychological well-being.

In Step 2, we estimated the most likely profile memberships from the posterior probabilities of the two-profile solution. Then, the profile-classification uncertainties in the latent profile membership were calculated for use in the next step. In Step 3, we tested the model presented in Figure 2. In this model, we examined the prediction of covariates (gender, years of education, and age) on profiles. Additionally, the latent profiles were used as a predictor of the SASS and PWB.

Table 2 shows the results of the latent logistic logit model. As Table 2 shows, only years of education significantly predicted the logit (*p* < 0.01). It was found that holding gender and age constant, the odds of being allocated in the Good configuration profile increased by 1.19 times for each additional year of education.

**Table 2:** Logistic regression.

Covariate	Estimate[OR]	S.E.	Estimate/S.E. (Wald)	*p*-value
Intercept	−2.557	1.247	−2.050	0.040
Gender	−0.015[0.985]	0.307	−0.049	0.961
Years of education	0.178[1.194]	0.062	2.887	0.004
Age	0.017[1.017]	0.020	0.846	0.397

OR, odds ratio; S.E., standard error; Study, years of education. Odds ratios are given in brackets; Feminine is the reference category for gender. Wald: Wald statistics. Effect of covariates on profiles.

**Figure 3:**
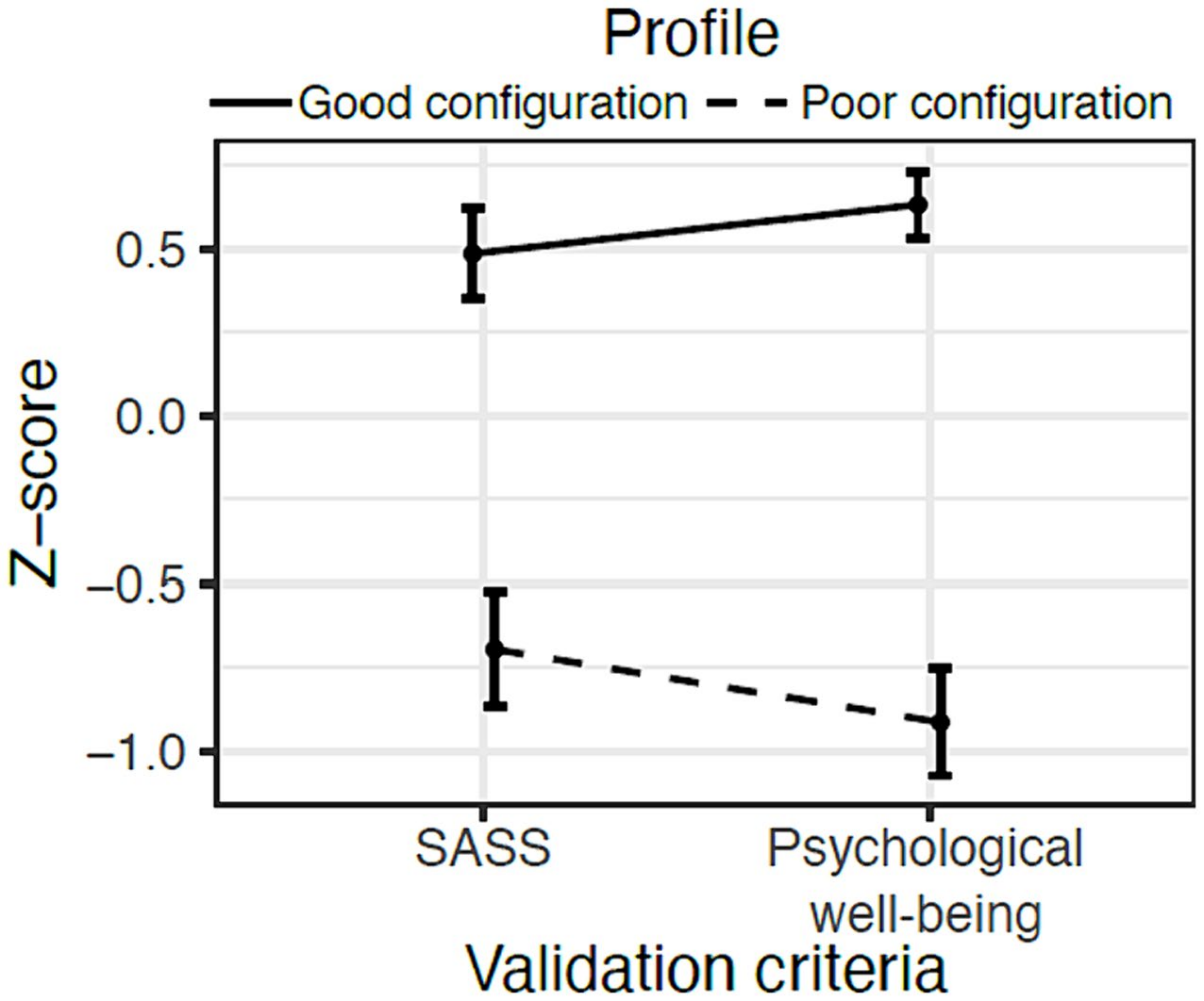
Means (dots) and 95% CIs (Bars) in SASS and PWB as a function of the profile. Bars represent 95% CIs of the mean.

In Step 3, we also computed a test for mean differences across profiles, where the Good and Poor-configuration profiles differed significantly in the scores related to SASS (*Estimate_Mean-differences_* = 1.182, *SE* = 0.111, *Wald* = 10.652, *p* = 0.000) and PWB (*Estimate_Mean-differences_* = 1.548, *SE* = 0.092, *Wald* = 16.890, *p* = 0.000). Figure 3 shows the profile-specific means and 95% CIs of the people’s scores in SASS and PWB as a function of the two profiles. The Good configuration profile scored significantly higher than the Poor configuration profile in SASS and PWB.

### 4.2 Machine-learning results

**Figure 4:**
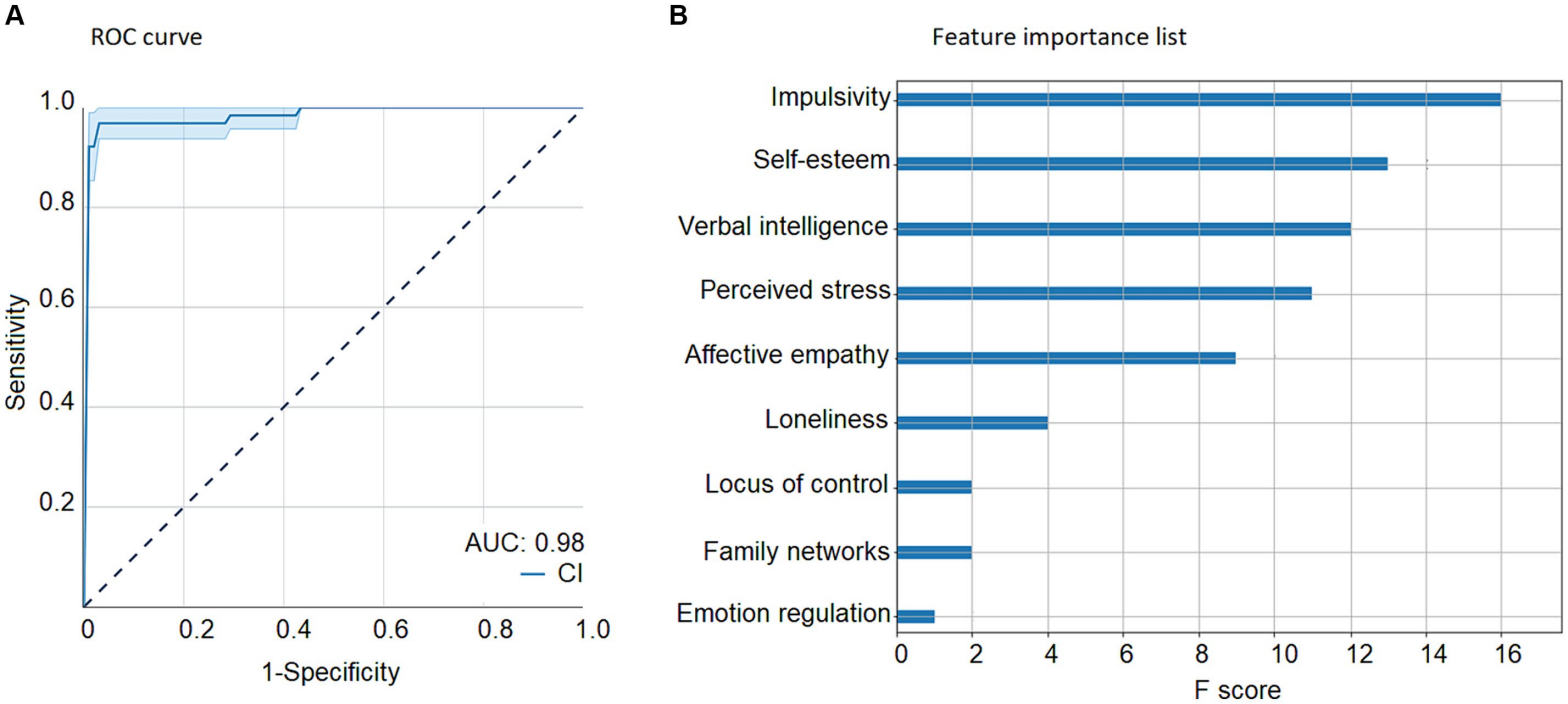
Machine-learning results with the full set of features and progressive feature elimination. Machine-learning results with the full set of features. **(A)** poor vs. good configuration ROC curve, indicating sensitivity (true positive rate) and 1 – specificity (false positive rate), while calculating the area under the curve (blue) and its confidence interval (light blue). Results show an AUC of 0.98. **(B)** Poor configuration vs. good configuration feature importance list of the most relevant features for the classification, with the impulsivity variable as the most important feature, followed by self-esteem, verbal intelligence, perceived stress, affective empathy, loneliness, locus of control, family networks and emotion regulation. AUC, Area under the curve; CI, Confidence interval.

We assessed the classification using the full set of features plus years of education because this variable predicted the likelihood of being classified into the profiles. The Poor vs. the Good configuration for psychosocial adaptation classification using the entire set of features yielded an AUC of 0.98 in the test-set (Figure 4A). The feature importance list (Figure 4B) for that classification resulted in the impulsivity variable as the most important feature, followed by self-esteem, verbal intelligence, perceived stress, affective empathy, loneliness, internal locus of control, family networks and finally, emotion regulation.

**Figure 5:**
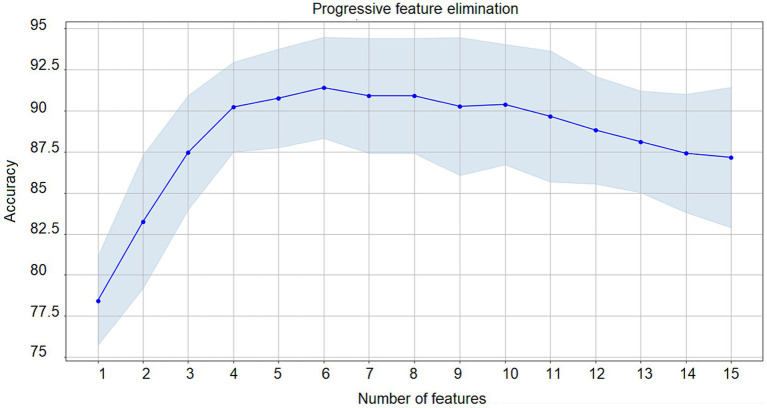
Progressive feature elimination. Progressive feature elimination. Iterative feature selection results starting from the full-set of features until an optimum number is obtained based on validation accuracy. For *N* = 5 the maximum accuracy (91.36%) was achieved with years of study, control of thoughts, perceived stress, impulsivity, self-esteem, and loneliness as the optimum feature set. Validation accuracy across folds (blue) and confidence interval (light blue).

Then, we performed the progressive feature elimination process to obtain the optimum set of features for that classification (Figure 5). After performing a 5-fold cross-validation on feature sets comprising different numbers of features, it has been found that a number of 6 features comprising self-esteem, impulsivity, loneliness, perceived stress, years of study, and control of thoughts yielded a maximum validation accuracy result of 91.36%.

**Figure 6:**
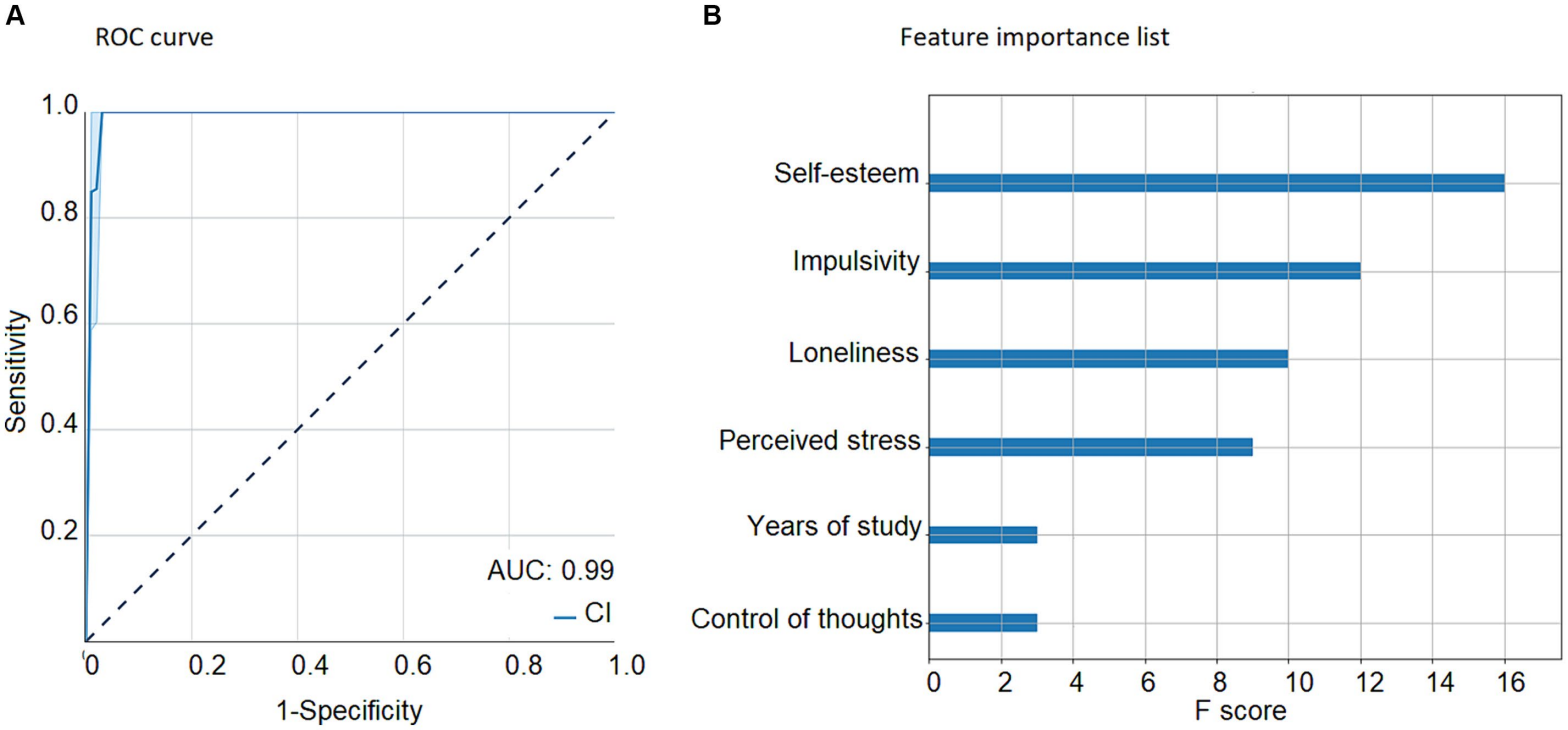
Machine-learning results after feature optimization. Machine-learning results after feature optimization. **(A)** poor vs. good configuration for psychosocial adaptation ROC curve, indicating sensitivity (true positive rate) and 1 – specificity (false positive rate), while calculating the area under the curve (blue) and its confidence interval (light blue). Results show an AUC of 0.99. **(B)** poor vs. good configuration feature importance list of the most relevant features for the classification, with the self-esteem variable as the most important feature, followed by impulsivity, loneliness, perceived stress, years of study, and control of thoughts. AUC, Area under the curve; CI, Confidence interval.

The Poor vs. Good configuration for psychosocial adaptation classification using the entire set of features yielded an AUC of 0.99 in the test-set (Figure 6A). The feature importance list (Figure 6B) for that classification resulted in the self-esteem variable as the most important feature, followed by impulsivity, loneliness, perceived stress, years of education, and control of thoughts.

## 5 Discussion

The aim of the present study was to identify individuals’ different latent profiles comprising the interaction of cognitive, emotional, and social variables among vulnerable adults who lived in impoverished areas in Santiago, Chile. Using a theoretically guided and evidence-based approach, we were able to identify critical variables, which predicted successful or unsuccessful psychosocial adaptation. Most previous research has adopted a variable-centered approach focusing on studying single variable outcomes. Although helpful, most of these studies did not include enough variables in complex models that allow them to control their interactions simultaneously (Fritz et al., 2018b). To fill this gap, the present study used LPA person-centered approach to identify different profiles of interacting variables and explore their association with sociodemographic predictors –gender, age, and years of education–and with different levels of psychosocial adaptation, operationalized as the by-product of SA and PWB. Moreover, we aimed to validate participants’ classification into the profiles through machine learning methods and, ultimately, identify the most important predictors of these different psychosocial adaptation paths through machine learning progressive feature elimination. We were able to identify two emerging profiles among the participants, which predicted successful or unsuccessful psychosocial adaptation. Nine out of the 11 initial selected indicators (LPA), namely emotion regulation, control of thoughts, impulsivity, locus of control, self-esteem, perceived stress, affective empathy, family networks, and loneliness were critical to differentiate between Good and Poor configuration for psychosocial adaptation. In contrast, classic predictors such as working memory capacity and verbal intelligence did not. In the following, we will discuss the results in terms of each of our research questions.

### 5.1 Identification of latent profiles of psychosocial adaptation among adults living in vulnerable contexts

LPA yielded two different profiles. These subgroups initially suggested a different configuration of cognitive, social and affective variables. Most people were assigned to Profile 1 (Good configuration for psychosocial adaptation). These people are characterized by good emotional, cognitive, and behavioral self-regulation, internal locus of control, high self-esteem, low-stress levels, low affective empathy, high family support, and low feelings of loneliness. In turn, people assigned to profile 2 (Poor configuration for psychosocial adaptation) showed the reverse pattern. According to previous literature, the configuration of scores shown by people allocated in Profile 1 (Good configuration for psychosocial adaptation) could facilitate psychosocial adaptation, whereas the configuration of Profile 2 (Poor configuration for psychosocial adaptation) scores could hinder psychosocial adaptation (Hobfoll, 2002; Huepe et al., 2011; Huepe and Salas, 2013; Meng et al., 2018; Fritz et al., 2018b; Gartland et al., 2019; Neely-Prado et al., 2019; Yule et al., 2019).

Most selected indicators (emotion regulation, control of thoughts, impulsivity, locus of control, self-esteem, perceived stress, affective empathy, family networks, and loneliness) could be considered as resources that significantly discriminated between good and poor psychosocial adaptation configuration profiles. The exception was cognitive variables: working memory capacity and verbal intelligence. The lack of significant differences between the profiles in these abilities runs against previous research that underlined the importance of working memory for promoting social adaptation and resilience to vulnerable contexts (Curtis and Cicchetti, 2003; Wingo et al., 2010; Levens et al., 2017; Neely-Prado et al., 2019; Bemath et al., 2020). Similarly, in regards to verbal intelligence, our results did not support those of previous research in children (Masten et al., 1999; Flouri et al., 2014) or adults (Neely-Prado et al., 2019). However, they align with (Brown et al., 2013), who found no strong support for the association of verbal intelligence and SA in a large longitudinal cohort of 1,603 boys living in poverty.

One possible explanation (Neely-Prado et al., 2019) is that previous research based on a variable-centered approach has tested the effect of these variables in isolation. In contrast, the person-centered approach adopted here, tested their effect in interaction with other variables. In fact, previously, some authors have pointed out that some variables could lose significance when tested simultaneously because of their cumulative or suppressing effects (Bonanno et al., 2011a) or because their effect could be modified or mediated by other factors (Bonanno and Diminich, 2013; Fritz et al., 2018b; Masten et al., 2021). Still, it could be possible that the presence of other socio-affective resources acting orchestrated could compensate for the lack of cognitive resources and that the effect of some resources could have a variable influence depending on time and circumstances as it is underscored by resources theories (Hobfoll, 2002). The interaction among resources and how it could suppress or enhance the effect of the others, is an important question that deserves more attention in future research.

Another result that deserves special attention is that people belonging to profile 1 (good configuration for psychosocial adaptation) showed lower levels of affective empathy than people belonging to profile 2 (poor configuration for psychosocial adaptation). Although empathy has been considered a motivator of social and prosocial behavior (Wolf et al., 2015; Decety et al., 2016; Eslinger and Long, 2016; Tomova et al., 2017) and as such would promote psychosocial adaptation, this may not be the case for all individuals living in chronically stressful contexts (Eisenberg et al., 2009; Cameron et al., 2019). In these contexts, feelings of grief or concern for others might overwhelm individuals, leading to personal distress that they would try to avoid. However, it is unclear why avoidance of personal distress might facilitate psychosocial adaptation. It could be that personal distress avoidance increases the feeling of PWB, which in turn promotes SA, but this is an open and controversial question to be explored in future research.

Regarding the predictors of the profiles, it is noteworthy that our results indicate that the probability of belonging to the profiles is not determined by sociodemographic factors such as participants’ gender or age. Instead, it is influenced by individuals’ possession and accessibility of psychological and social resources to confront adversity, along with the strategies they may employ to select, optimize, and compensate for these resources, as posited by resource theories (Hobfoll, 1989; Baltes, 1997; Hobfoll, 2002; Hobfoll et al., 2015; Holmgreen et al., 2017). Such findings are important because demographic variables reflect personal attributed not modifiable, whereas individual and social resources could be more easily modifiable by psychological or social interventions (see Lent, 2004 for a similar account). In contrast, another sociodemographic variable, higher educational level, predicts the probability of belonging to a good adaptation profile. These results align with previous studies showing positive correlation between the level of education and social adaptation (Neely-Prado et al., 2019) in similar populations as well as associations between low educational levels and social maladjustment (Groot and van den Brink, 2010; Livingston et al., 2010; Liu et al., 2021), fewer employment opportunities and insufficient income (Thompson and Dahling, 2019; Papadakis et al., 2020). Therefore, this result highlights the relevance of formal education not only to acquiring information, reading, or mathematical skills useful for life, but also to learning psychological skills that foster resilience (Masten and Cicchetti, 2016).

Additionally, we internally validated the classification into Good and Poor configurations for adaptation profiles obtained from LPA through machine learning methods, obtaining an excellent classification accuracy with the initial set of indicators plus years of education. Therefore, both LPA and machine learning classification results point out that the theoretical selection of most of the social and emotional indicators was valid for discriminating between different pathways of psychosocial adaptation within the same context of vulnerability (Anthony, 2008; Buckner et al., 2009; Cicchetti, 2013; Nguyen and Joel Wong, 2013; Meng et al., 2018; Fritz et al., 2018b; Gartland et al., 2019; Neely-Prado et al., 2019).

### 5.2 Critical variables aiding the classification of psychosocial adaptation profiles

After validating the profiles obtained from LPA, we conducted machine learning based progressive feature elimination procedure to obtain the optimal set of features for classifying into good and poor configuration profiles. It showed a maximum validation accuracy with only 6 of the 11 indicators: self-esteem, impulsivity, loneliness, perceived stress, years of education, and control of thoughts.

That is, although most of the initial indicators differentiated the two profiles of psychosocial adaptation, we obtained mostly the same accuracy with only six of them, which is important not only in terms of parsimony in the explanation, but also for the design of future psychosocial interventions with these populations.

### 5.3 Latent profiles and their association with psychosocial adaptation

We used the SASS and the P PWB scales as pointers for psychosocial adaptation to validate the two identified profiles. Crucially, the Good configuration for psychosocial adaptation profile scored significantly higher on the SASS and PWB scales. These results align with the predicted association between SA and PWB (Lent, 2004; Blanco and Díaz, 2005; Masten et al., 2021), as both profiles were associated with SA and PWB in the same direction. However, given that the constructs are not identical, we suggest that in the Good configuration profile, some features selected by the machine learning analysis (high scores in self-esteem and low control of thoughts) could be more related to PWB, while the remaining features (low scores in impulsivity and low perceived stress) promote better adjustment in social relationships, a greater sense of belonging and contribution to society, aspects mainly linked SA. Taken together, all these correlational results only suggest a possible pathway of influence, not the mechanisms through which they exert their influence, a matter beyond the scope of our current objectives and left open for future research.

Although the comparison with previous studies followed a variable-centered approach is not straightforward, globally the results agreed with those that underlined that not all people living in impoverished environments develop adverse outcomes. Instead, having personal and social resources could help people cope with adversity and stress (Baltes, 1997; Hobfoll, 2002; Bonanno and Diminich, 2013). Among the resources that could act as protectors of maladaptive outcomes, in agreement with previous research, are high self-esteem (Kesting et al., 2013; Orth and Robins, 2014; Pu et al., 2017; Neely-Prado et al., 2019), behavioral and cognitive self-regulation (Aspinwall and Taylor, 1997; Buckner et al., 2009; Flouri et al., 2014), as well as adequate levels of education (Groot and van den Brink, 2010; Livingston et al., 2010; Neely-Prado et al., 2019; Thompson and Dahling, 2019; Papadakis et al., 2020; Liu et al., 2021). Conversely, loneliness (Dumont and Provost, 1999; Anthony, 2008; Fritz et al., 2018b; Gartland et al., 2019) and high perceived stress levels (Neely-Prado et al., 2019; Masten et al., 2021) could act as risk factors for maladjustment. Outstandingly, these most important indicators for psychosocial adaptation are very much the same as those identified in the Kauai longitudinal study (Werner, 1996, 2015). Their results found that resilient adolescents show more flexibility in dealing with stress, together with high cognitive abilities, good impulse control, a high reflective cognitive style, more internal locus of control, and higher self-esteem.

These results underlined the importance of studying a constellation of orchestrated variables (Holahan et al., 1999; Hobfoll, 2002; Bonanno and Diminich, 2013). Our results pointed out that in confronting adversity, most people showing adaptive strategies used an array of dynamic interacting resources (Bonanno and Diminich, 2013). The resources and strategies available in this context are varied, encompassing both internal and external factors that configure different pathways for psychosocial adaptation (Hobfoll, 2002; Masten et al., 2021). Our results pointed out that internal resources such as high self-esteem, stress resistance, and the ability to self-regulate behavior and cognition can assist individuals in maintaining a positive outlook, finding creative solutions to challenges, and sustaining motivation and hope during difficult times. External resources including social support and education, also play a crucial role. Social support, in particular, has been identified as pivotal in individuals’ ability to cope with adversity, providing emotional solace, practical guidance, and material aid.

Moreover, it is plausible that certain resources may causally engender other mechanisms (Holahan et al., 1999; Masten et al., 2021), create positive and avoid negative cascades, or exhibit compensatory or protective effects in the face of adversity (Hobfoll, 2002; Masten et al., 2021). For instance, some evidence suggests the existence of social buffering of the hypothalamic–adrenal–pituitary (HPA) system, wherein social support can moderate stress reactions across development (Hostinar et al., 2014; McEwen, 2020; Masten et al., 2021). Other studies suggested that effective parenting may act as a buffer for limiting the harm posed by early adversity, interrupting negative cascades and promoting positive ones (Masten and Kalstabakken, 2018). Moreover, there is evidence indicating dynamically modeled associations within broader networks of interconnected variables or resources. Within these networks (e.g., Fritz et al., 2018a; Kalisch et al., 2019), protective factors may be activated by stressors and dynamically operate to either weaken or strengthen the interconnectedness of resources, which may vary over time and populations. Unfortunately, the present design does not allow us to test these causal relations and mechanisms among resources, nor does it establish specific pathways of interaction among resources that could be multiple and not linear over the course of lifespan development (Masten et al., 2021). Still, the causal relationships among the identified resources, the strategic use of them, and the potential compensatory or protective effects are key questions to be addressed in future research through other methodologies as, for instance, path analysis, growth mixture modeling, or network modeling.

Moreover, it is essential to note that not all resources are equally stable and available throughout the lifespan (Schwartz, 2000), and that sensitivity to chronic stress is variable and determined by behavioral and personality variables (Werner, 1996, 2015; Yuen et al., 2009; McEwen, 2012; Cicchetti, 2013; De Minzi and Lemos, 2014; Meng et al., 2018); as well as by differential susceptibility or sensibility to context (e.g., Ellis et al., 2011). Our results are only a snapshot of a particular resource constellation at a given time which could fluctuate as life circumstances change. In this sense, given that adaptation is a balancing process between resilience and allostatic load (Meng et al., 2018; Yule et al., 2019), it could be interesting to explore in future research using longitudinal designs and multiple methods for modeling interactions, whether the potential protective effect of self-esteem and self-regulation fluctuates under changing circumstances of other less stable resources such as social support or perceived stress. This aspect is crucial for comprehending how individuals optimize available resources or compensate for their absence when faced with varying life circumstances, as emphasized by the SOC theory (Baltes, 1997), and for understanding how individuals interact with and adapt to the numerous internal and external systems and processes, as proposed by the dynamic systems perspectives of resilience (e.g., Masten et al., 2021).

## 6 Limitations

A possible limitation of this study is the use of self-reported measures; therefore, social desirability could have affected the scores. However, given that we obtained two different and consistent profiles and our reliable measures, we are reasonably confident in the results. Another possible limitation is that these results are context-dependent and, therefore, do not extrapolate to other populations living in similar poverty contexts, where unlike in Chile, access to public education is not guaranteed for all the population. A mitigating aspect of the approach is that it can be used for previous datasets and look for convergent or divergent results to strengthen the conclusions.

## 7 Conclusion

We confirm that social, cognitive, and affective variables, such as high self-esteem and cognitive and behavioral self-regulation, low levels of stress, level of education, and social support, act coordinately and are crucial for differentiating diverse pathways of psychosocial adaptation. This points out that, even in objectively bad living conditions, people with more social, cognitive, and emotional resources could have better expectations of experiencing positive outcomes (Hobfoll, 2002). The optimal set of resources identified has important implications for designing psychosocial policy in vulnerable contexts through the enhancement of protective factors like self-esteem and self-regulation, which could aid in reducing the impact of some risk factors such as perceived stress and loneliness. Moreover, it is worth noting that the adequate access to public education, even in an impoverishing context, acts as a protective factor against psychosocial maladjustment.

## Data availability statement

The raw data supporting the conclusions of this article will be made available by the authors, without undue reservation.

## Ethics statement

The studies involving humans were approved by Universidad Adolfo Ibañez ethics committee. The studies were conducted in accordance with the local legislation and institutional requirements. The participants provided their written informed consent to participate in this study.

## Author contributions

NC: Conceptualization, Writing – original draft, Writing – review & editing. OR-V: Formal analysis, Methodology, Writing – original draft. SM: Formal analysis, Methodology, Writing – review & editing. JM-S: Writing – review & editing. DH-A: Writing – review & editing. VS: Writing – review & editing. DF-O’B: Project administration, Writing – review & editing. AI: Funding acquisition, Writing – review & editing. TB: Writing – review & editing. DH: Conceptualization, Funding acquisition, Supervision, Writing – original draft.

## Footnotes

^1^Retrieved from http://www.registrosocial.gob.cl/que-es#top
