# Supplementary material for: Cognitive, emotional, and social factors promoting psychosocial adaptation: a study of latent profiles in people living in socially vulnerable contexts
Source: Front Psychol. 2024 Apr 12;15:1321242. doi: 10.3389/fpsyg.2024.1321242 (PMC11050042; doi:10.3389/fpsyg.2024.1321242)
Supplement: Supplementary file 1 [file Data_Sheet_1.PDF]

Section A. Materials

Self-regulation.

Metacognition questionnaire 30 (MCQ-30) - control of thoughts (Wells & Cartwright-Hatton, 2004). This questionnaire is made up of 30 items and has five dimensions: (1) positive beliefs about worries, which measures how useful the person thinks the worry is; (2) negative beliefs about worry, which measure how complex and uncontrollable the subject considers worry to be; (3) cognitive confidence, which measures the confidence that the subject has in their attention and memory processes; (4) beliefs about the need for control, which measures the subject's need to control and/or eliminate specific thoughts; and (5) cognitive self-awareness, which measures the subject's tendency to monitor attention to their thoughts (Ramos-Cejudo, Salguero, & Cano-Vindel, 2013). We chose the need to control thoughts subscale (items 19-24). The score ranges from 1 ("totally disagree") to 4 ("totally agree). A higher score indicates higher levels of pathological metacognition. The scale's reliability was α =.89 (Ramos-Cejudo et al., 2013). The reliability for the subscale in this sample was α =.76

Emotional Regulation Questionnaire (ERQ)– Cognitive re-evaluation (Gross & John, 2003). It is a 10-item self-report scale that measures two dimensions: "cognitive reappraisal" and "emotional suppression". It is suggested that both dimensions correspond to different emotional regulation strategies. We chose cognitive re-evaluation. The response options are structured on a Likert-type scale from 1 ("strongly disagree") to 7 ("strongly agree"). The scale's reliability was α =.79 (Cabello, Salguero, Fernández-Berrocal, & Gross, 2013). The reliability of the Cognitive re-evaluation subscale for this sample was α =.77.

Barrat impulsivity scale (BIS-11) (Patton, Stanford, & Barratt, 1995). It is a 30-item self-application questionnaire that measures levels of impulsivity through three dimensions: "cognitive impulsivity", "motor impulsivity" and "unplanned impulsivity". It is validated in the Chilean population (Salvo & Castro, 2013). The scale consists of 30 items with response options ranging from 1 to 4 on a Likert-type scale from 1 ("rarely or never") to 4 ("always or almost always"). The totals per dimension and the scale's total score are obtained by adding the responses. The reliability of the scale was (α = .77) (Salvo & Castro, 2013). The reliability of this sample was α = .73.

Self-esteem. Rosenberg self-esteem scale. This scale consists of 10 items and globally assesses self-esteem, that is, the value that people have of themselves (Rosenberg, 1965). The Rosenberg Self-Esteem Scale is a self-administered test in which the participant

must mark the correct alternative for each slogan. According to Rosenberg (1965), the scores range from 10 (low self-esteem) to 40 (high self-esteem). They are assigned a score between 1 ("strongly disagree") and 4 points ("strongly agree"). Items 3, 5, 8, 9, and 10 must be reversed (the higher the agreement, the lower the score). The reliability of the scale was α = .75 (Rojas-Barahona, Zegers, & Förster, 2009).The reliability for this sample was α = .86.

Social Support

UCLA Loneliness Scale (Russell, 1996). It is composed of 8 items that seek to measure the subject's experience in relation to loneliness and social support. This scale is self-administered and consists of 20 items, 10 of a positive nature and 10 of a negative nature. Each item is evaluated through "O", which indicates "I often feel this way", "S", which indicates "I sometimes feel this way"; "R", which indicates "rarely feel this way" and "N", which indicates "I never feel this way". Then, all the "O" are evaluated with 3 points, the "S" with 2 points, the "R" with 1 point, and the "N" with 0 point. The higher the score, the more loneliness. The reliability of the scale ranged from α = .89 to α = .92 in different populations. The reliability of this sample was α = .84.

Lubben Social Network Scale–Revised (LSNS-R) – family network (Lubben, 1988). It consists of 12 items that evaluate social networks through two subscales: family networks and social networks, including friends. Each subscale has a maximum of 30 points, leading to a maximum total score of 60 points. The LSNS-R is self-administered and has 12 items from 0 to 5, being 5 high frequencies. This score is added, and the lower the total result, the fewer social networks the evaluated has. The reliability of the scale was α = .90 (Wells, 2010). The family network subscale for this sample was α = .78. The family networks scale measures the type and frequency of interaction with family members and how an individual perceives the availability of family members (Lubben, 1988). Higher scores on this observed variable are related to better mental and physical health while lower scores are linked to depressive symptoms and a diminished quality of life (Jang, et al., 2022).

Internal locus of control.

Attributional style – Internality. (Levenson, 1974). This scale measures the locus of control. This instrument consists of 24 items, and it evaluates the attributions that people make on the circumstances in three dimensions: "chance", "powerful others", and "internality" the last one is the dimension used in this study. We chose the internality

dimension which refers to the frequency in which the person attributes that situations in life are the product of their own actions and decisions. It is a self-administered test in which each participant responds using a Likert scale of 6 options, which is scored from 1 ("completely disagree") to 6 ("completely agree"). The scores vary between 0 and 8. The higher the score, the higher internality in the attributional style. The reliability of internality dimension of the scale was $\alpha = .71$ (Manso-Pinto & Ruggieri-Vega, 1985), and for this sample was $\alpha = .70$.

Empathy.

The interpersonal reactivity index IRI (Davis, 1983). It is a self-report instrument of 28 items that address four empathy dimensions. "Perspective taking" measures whether the person tends to incorporate the point of view of others on a day-to-day basis; "Fantasy" refers to the tendency to internally adopt the way of thinking, feeling and acting of fictional characters as if the person themselve was the character; "Empathic concern" addresses the tendency to feel warmth, compassion and concern for others; and "Personal discomfort" refers to the tendency to feel uncomfortable in response to the emotions of other. We chose the "Affective empathy" dimension composed of "empathic concern" and "personal discomfort". The answers are based on a Likert scale that ranges from 0 ("it does not describe me well") to 4 ("it describes me very well"). The score for each dimension and for the total of the scale is obtained from the sum of the responses, with 28 being the maximum score per dimension. The reliability of the scale was $\alpha = .70$ (Fernández, Dufey, & Kramp, 2011). The reliability of this sample for affective empathy dimension was $\alpha = .62$.

Stress.

Perceived stress scale (PSS). This scale seeks, through 14 items, to measure the level of stress generated by daily events in people's lives (Cohen, Kamarck, & Mermelstein, 1983). This self-administered scale consists of 14 items that describe the level of perceived stress in the last month. The evaluated person answers from 0 to 4 on a Likert scale on the frequency of the situations described in the items related to them (Remor, 2006). The answers are 0 = "never", 1 = "almost never", 2 = "once in a while", 3 = "often", 4 = "very often", and each number corresponds to the score generated by each answer. The higher the score obtained, the higher the level of perceived stress. The reliability of the scale was $\alpha = .81$ (Remor, 2006). The reliability of this sample was also $\alpha = .81$.

Cognitive abilities

Working Memory. WAIS IV – digit span backward (Rosas et al., 2014)**.** This scale consists of 8 items. It measures the functioning of verbal working memory and the coding of short-term memory. People are asked to repeat in reverse order the sequence of numbers that is dictated. For each attempt, 1 point is awarded if the answer is correct, and 0 points if it is incorrect or the person does not respond within approximately 30 seconds. The test is suspended in case the evaluated obtains zero points in two attempts of an item. The total score is obtained from the sum of the scores in each attempt and a total "span" score refers to the number of information units that the participant is able to retain in each section (Amador, 2013). The reliability of the scale was α = .74 (Rosas et al., 2014). The reliability of this sample, using split-half methods (Spearman-Brown coefficient), was α = .60.

Verbal intelligence. WAIS IV – vocabulary (Wechsler, 2003). It consists of 30 items (3 to practice and 27 experimental). Practice items are scored from 0 to 1 points and experimental items are scored from 0 to 2 points. The stimuli are presented in the form of a question ("Tell me what it means...") or just mentioning the word in the more advanced items. The questions can be repeated as many times as necessary, starting with item 5. If the subject does not obtain the maximum score on items 5 or 6, the previous items must be administered in reverse order until perfect scores are obtained in two consecutive items. If the person answers items 5 and 6 correctly, they are given the maximum score in items 1, 2, 3, and 4. When the examinee reports a good understanding of the word, he is awarded 2 points. If the examinee shows a correct answer but poor in content of the meaning of the word, they get 1 point, while they get 0 points if giving a wrong answer. The scale is suspended after three consecutive scores of 0. A wide range of specific examples for each item and their scores can be found in the instrument manual. The maximum score is 55 which is the sum of the score obtained in each item, however it is necessary to scale the scores according to the age of the subject. The reliability of the scale was α = .91 (Rosas et al., 2014) The reliability of this sample, using split-half methods (Spearman-Brown coefficient), was α = .86.

Psychosocial adaptation.

Social Adaptation Self-Regulation Scale (SASS) (Bosc, Dubini, & Polin, 1997). It is composed of 21 items devoted to measure social motivation and behaviour. Through four dimensions the scale evaluates relationships outside the family, work and leisure, socio-cultural interests and family relationships and behavioral strategies (Bobes et al., 1999). People has to mark the degree of agreement with each item in a scale from 0 (nothing)

to 3 (a lot). We used the total score of the scale. Its reliability was α = .78. The reliability of this sample was α = .73

Psychological Well-being. Psychological Well-being scale (PWB) (Ryff & Singer, 2006). It is composed of 29 items for measuring six dimensions: self-acceptance, autonomy, positive relationships with others, purpose in life, mastery of the environment, and personal growth. It is a Likert scale ranging from 1 (completely disagree) to 6 (completely agree) Reliability of the scale ranged from α = .68 to α = .83 (Díaz et al., 2006). The reliability of the sample was α = .91.

Psychosocial adaptation was operationalized as the by-product of Social adaptation (SA) and Psychological well-being (PWB). However, SA and PWB are related but distinct constructs. SA, measured by the Social Adaptation Self-evaluation Scale (SASS), examines the quality of non-family relationships (N_FR), work and leisure (W_L), sociocultural interests', (INT) and family relationships (FAM). Psychological well-being as proposed by Ryff and Singer (2006) is a broader construct that involves areas such as positive relationships (R_POS), self-acceptance (S_A), autonomy (AU), environmental mastery (E_M), personal growth (P_G), and purpose in life (P_L). To understand the relation between SA and PWB we conducted a confirmatory factor analysis for testing the following hypotheses: 1) the first hypothesis resembled a model in which both factors are correlated (see Figure S1 model A); 2) the second hypothesis evaluated a model in which R_POS, FAM, and INT are the variables explaining part of the relationship between SA and PWB (see Figure S1 model B); and 3) the last hypothesis examined whether FAM and R_POS are the key variables underlying the latter relationship (see Figure S1 model C). Model A shows a strong correlation between SA and PWB which confirms that both scales shared a 53,29% of the variance. . The results of model B indicate that the inclusion of the cross-loadings R_POS, FAM, and INT decrease the correlation between these factors from .73 to .62. However, in model B, the regression path from P_WB to INT was not different from zero. In model C, FAM and R_POS cross-loadings were significant, and although the correlation pattern decreased to .54 it was still significant. The evaluation of this series of models suggests that FAM and R_POS, although important, are not the main variables explaining the correlation found in Model A between SASS and PWB. On the contrary, the moderate association between the scales in Model C (only share a 29% of the variance) reflects the co-occurrence of different factors.

Supplementary analysis: Confirmatory factor analysis

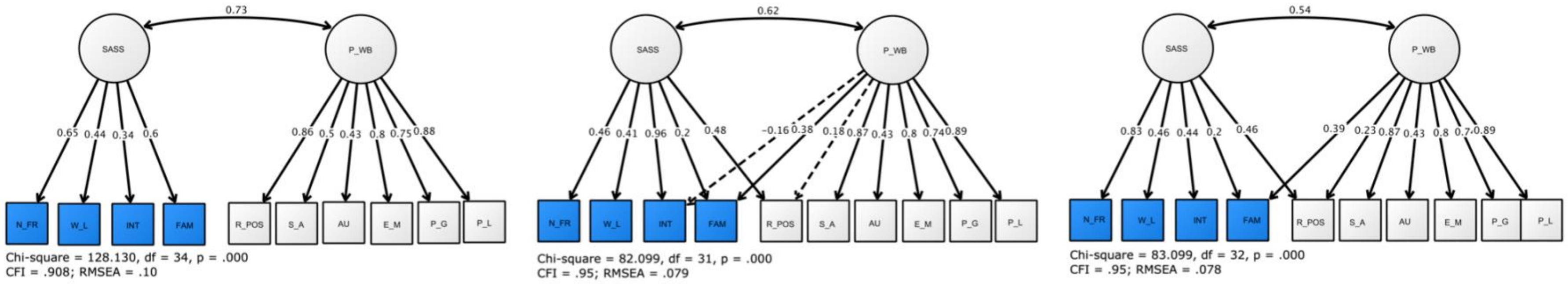

Figure S1. Note. N_FR: non-family relationship, W_L: work and leisure; INT: sociocultural interests'; FAM: Family relationships and behavioral strategies; R_POS: positive relationships; S_A: self-acceptance; AU: autonomy; E_M= environmental mastery; P_G: personal growth; P_L: purpose in life; SA: Social adaptation; P_WB: Psychological well-being; df: degree of freedom; CFI: comparative fit index; RMSEA: Root Mean Square Error of Approximation. Based on CFI and RMSEA the model A to the data was acceptable, while the fit of Model B a C to the data was good.

Section B. Statistical analysis

Criteria used to select models in LPA

The AIC and BIC criteria are based on the model log likelihood and penalty terms related to model complexity (both indices) and sample size (BIC). In these criteria, smaller values indicate the preferred model. Between these indices, simulation studies show that the BIC performs the best (Nylund, 2007; Nylund, Asparouhov, & Muthén, 2007). The Entropy is a summary measure whose value ranges from 0 to 1 where the latter value indicates a perfect classification. According to Clark & Muthén (2009), an Entropy value of .80 or higher is good and values between .60 and .80 are also seen as appropriate (Jung & Wickrama, 2008). The BLRT compares one model with k profiles with a model with k + 1 profiles on their model fit. This nested model test uses bootstrap samples to evaluate the distribution of the log-likelihood difference test statistic between a model with k profiles and a model with k + 1 profiles. A p-value that is not significant for this test indicates that the k model is preferred for the BLRT.

The XGBoost algorithm

The XGBoost algorithm is a Gradient Boosting Machines (GBM) implementation that provides parallel computation tree boosting, enabling fast and accurate predictions, and advanced regularization techniques to avoid overfitting. GBMs are based on the gradient boosting technique, in which ensembles of decision trees iteratively attempt to correct the classification errors of their predecessors by minimizing a loss function (i.e., a function representing the difference between the estimated and true values) pointing in the negative gradient direction (Mason, Baxter, Bartlett, & Frean, 1999). This algorithm has been proven successful in several diagnostic applications (Behravan et al., 2018; Moguilner et al., 2021; Torlay, Perrone-Bertolotti, Thomas, & Baciu, 2017; Zheng, Yuan, & Chen, 2017). When compared to other GBM algorithms, XGBoost provides regularized boosting, helping to reduce overfitting and thus providing more generalizable results (Torlay et al., 2017).

The XGBoost has several hyperparameters, such as the learning rate, the minimum loss reduction required to make a further partition of a leaf node, the maximum depth of a tree, the maximum number of leaves, and the regularization weights. In order to choose the best parameters for the classification in this high dimensional hyperparameter space, we used Bayesian Optimization (Feurer & Hutter, 2019; Zeng & Luo, 2017). This state-of-the-art optimization framework demonstrated wide applicability to different problem settings. This is an iterative algorithm with two key ingredients: a probabilistic surrogate model and an acquisition function to decide

which point to evaluate next. At each step, a new point of the hyper-parameter space to explore is selected to be the maximum of an activation function of the prior knowledge and the uncertainty. As this optimization progresses, the chances of finding a better solution increase. Compared to other techniques such as the grid-search which is undermined by issues of dimensionality or random-search (where each guess is independent from the previous run), the Bayesian optimization algorithm is fast to compute, enabling a thorough optimization of the hyper-parameters.

Table S1

Supplementary table S1

Descriptive statistics of measured variables

	Profiles	*N*	MEAN	SD	MIN	MAX	RANGE	SE
Years of education	Good configuration	151	14.12	2.73	6	21	15	0.22
	Poor configuration	100	13.02	2.97	5	22	17	0.30
Age	Good configuration	153	31.58	7.80	18	46	28	0.63
	Poor configuration	101	31.71	8.22	18	46	28	0.82
Locus of control	Good configuration	152	39.61	5.19	21	48	27	0.42
	Poor configuration	99	36.14	5.77	19	48	29	0.58
Emotion regulation	Good configuration	153	31.74	6.71	11	42	31	0.54
	Poor configuration	97	27.70	7.28	8	42	34	0.74
Self-esteem	Good configuration	150	35.31	3.17	28	40	12	0.26
	Poor configuration	100	27.32	4.30	15	37	22	0.43
Perceived stress	Good configuration	153	22.59	5.02	11	35	24	0.41
	Poor configuration	101	29.90	4.89	20	42	22	0.49
Impulsivity	Good configuration	153	64.10	8.02	44	90	46	0.65
	Poor configuration	101	74.04	8.83	53	96	43	0.88
Control thoughts	Good configuration	152	13.16	3.96	6	24	18	0.32
	Poor configuration	100	15.29	4.44	6	24	18	0.44

	Profiles	*N*	MEAN	SD	MIN	MAX	RANGE	SE
Loneliness	Good configuration	153	3.16	2.46	0	11	11	0.20
	Poor configuration	100	6.68	2.85	2	11	9	0.28
Family networks	Good configuration	153	19.92	4.76	5	30	25	0.38
	Poor configuration	100	15.25	5.53	2	24	22	0.55
Affective empathy	Good configuration	152	29.78	6.61	15	44	29	0.54
	Poor configuration	99	32.75	7.38	16	52	36	0.74
Working memory	Good configuration	151	7.39	2.16	3	13	10	0.18
	Poor configuration	98	7.12	2.27	3	14	11	0.23
Verbal intelligence	Good configuration	148	27.82	8.29	10	44	34	0.68
	Poor configuration	95	24.74	9.09	10	50	40	0.93
Social adaptation	Good configuration	153	45.10	5.48	30	56	26	0.44
	Poor configuration	101	38.60	6.28	19	51	32	0.62
Psychological well-being	Good configuration	153	147.45	15.36	98	174	76	1.24
	Poor configuration	100	116.94	18.62	77	169	92	1.86

Note. The first column shows sociodemographic, profile features and criterion variables measured in this study. The second column shows the social adaptation profile characterized by a good/ poor configuration of social adaptation features. The following columns indicate the number of cases, mean, standard deviation, minimum/maximum scores, range and standard error respectively.

References

Amador, J. A. (2013). *Escala de inteligencia de Wechsler para adultos-IV (WAIS-IV).*

Behravan, H., Hartikainen, J. M., Tengström, M., Pylkäs, K., Winqvist, R., Kosma, V.-M., & Mannermaa, A. (2018). Machine learning identifies interacting genetic variants contributing to breast cancer risk: A case study in Finnish cases and controls. *Scientific Reports*, *8*(1), 13149. https://doi.org/10.1038/s41598-018-31573-5

Bobes, J., González, M. P., Bascarán, M. T., Corominas, A., Adán, A., Sánchez, J., & Such, P. (1999). [Validation of the Spanish version of the social adaptation scale in depressive patients]. *Actas espanolas de psiquiatria*, *27*(2), 71–80.

Bosc, M., Dubini, A., & Polin, V. (1997). Development and validation of a social functioning scale, the social adaptation self-evaluation scale. *European Neuropsychopharmacology*. https://doi.org/10.1016/S0924-977X(97)00420-3

Cabello, R., Salguero, J. M., Fernández-Berrocal, P., & Gross, J. J. (2013). A Spanish adaptation of the Emotion Regulation Questionnaire. *European Journal of Psychological Assessment*, *29*(4), 234–240. https://doi.org/10.1027/1015-5759/a000150

Clark, S. L., & Muthén, B. (2009). *Relating latent class analysis results to variables not included in the analysis*. Los Angeles, California, USA.

Cohen, S., Kamarck, T., & Mermelstein, R. (1983). A global measure of perceived stress. *Journal of Health and Social Behavior*. https://doi.org/10.2307/2136404

Davis, M. H. (1983). Measuring individual differences in empathy: Evidence for a multidimensional approach. *Journal of Personality and Social Psychology*. https://doi.org/10.1037/0022-3514.44.1.113

Díaz, D., Rodríguez-Carvajal, R., Blanco, A., Moreno-Jiménez, B., Gallardo, I., Valle, C., & van Dierendonck, D. (2006). [Spanish adaptation of the Psychological Well-Being Scales (PWBS)]. *Psicothema*, *18*(3), 572–577.

Fernández, A. M., Dufey, M., & Kramp, U. (2011). Testing the psychometric properties of the Interpersonal Reactivity Index (IRI) in Chile: Empathy in a different cultural context. *European Journal of Psychological Assessment*, *27*(3), 179–185. https://doi.org/10.1027/1015-5759/a000065

Feurer, M., & Hutter, F. (2019). Hyperparameter optimization. *In Automated Machine Learning*, 3–33.

Gross, J. J., & John, O. P. (2003). Individual differences in two emotion regulation processes: implications for affect, relationships, and well-being. *Journal of Personality and Social Psychology*, *85*(2), 348.

Jang, Y., Powers, D.A., Park, N.S., Chiriboga, D.A., Chi, I., Lubben, J. (2022). Performance of an Abbreviated Lubben Social Network Scale (LSNS-6) in Three Ethnic Groups of Older Asian Americans. *Gerontologist 9*,62(2), 73-81. doi: 10.1093/geront/gnaa156. PMID: 33021635.

Jung, T., & Wickrama, K. A. S. (2008). *An introduction to latent class growth analysis and growth mixture modeling. Soc Personal Psychol Compass 2: 302–317.*

Levenson, H. (1974). Activism and Powerful Others: Distinctions Within the Concept of Internal-External Control. *Journal of Personality Assessment*. https://doi.org/10.1080/00223891.1974.10119988

Lubben, J. E. (1988). Assessing social networks among elderly populations. *Family and Community Health*. https://doi.org/10.1097/00003727-198811000-00008

Manso-Pinto, J. F., & Ruggieri-Vega, E. A. (1985). Perceived Locus of Control Among Chilean University Students. *The Journal of Social Psychology*, *125*(6), 783–785. https://doi.org/10.1080/00224545.1985.9713556

Mason, L., Baxter, J., Bartlett, P., & Frean, M. (1999). Boosting algorithms as gradient descent. *Advances in Neural Information Processing Systems*, *12*.

Moguilner, S., Birba, A., Fino, D., Isoardi, R., Huetagoyena, C., Otoya, R., … García, A. M. (2021). Multimodal neurocognitive markers of frontal lobe epilepsy: Insights from ecological text processing. *NeuroImage*, *235*, 117998. https://doi.org/10.1016/j.neuroimage.2021.117998

Nylund, K. (2007). *Latent transition analysis: Modeling extensions and an application to peer victimization*. Citeseer.

Nylund, K., Asparouhov, T., & Muthén, B. (2007). Deciding on the number of classes in latent class analysis and growth mixture modeling: A Monte Carlo simulation study. *Structural Equation Modeling: A Multidisciplinary Journal*, *14*(4), 535–569.

Patton, J. H., Stanford, M. S., & Barratt, E. S. (1995). Factor structure of the Barratt impulsiveness scale. *Journal of Clinical Psychology*, *51*(6), 768–774.

Ramos-Cejudo, J., Salguero, J. M., & Cano-Vindel, A. (2013). Spanish version of the

meta-cognitions questionnaire 30 (MCQ-30). *Spanish Journal of Psychology*. https://doi.org/10.1017/sjp.2013.95

Remor, E. (2006). Psychometric properties of a European Spanish version of the Perceived Stress Scale (PSS). *Spanish Journal of Psychology*. https://doi.org/10.1017/S1138741600006004

Rojas-Barahona, C. A., Zegers, B., & Förster, C. E. (2009). La escala de autoestima de Rosenberg: Validación para Chile en una muestra de jóvenes adultos, adultos y adultos mayores. *Revista Médica de Chile*, *137*(6), 791–800.

Rosas, R., Tenorio, M., Pizarro, M., Cumsille, P., Bosch, A., Arancibia, S., ... Zapata-Sepulveda, P. (2014). Estandarización de la Escala Wechsler de Inteligencia Para Adultos-Cuarta Edición en Chile. *Psykhe*, *23*(1), 1–18. https://doi.org/10.7764/PSYKHE.23.1.529

Rosenberg, M. (1965). Rosenberg self-esteem scale (RSE). *Measures Package*. https://doi.org/10.1177/0145445507302037

Russell, D. (1996). Ucla Loneliness Scale Version 3 (description of Measure). *Journal of Personality and Social Psychology*.

Ryff, C. (2014). Psychological well-being revisited: Advances in the science and practice of eudaimonia. *Psychotherapy and Psychosomatics, 83(1)*, 10-28. https://doi.org/10.1159/000353263

Ryff, & Singer, B. H. (2006). Best news yet on the six-factor model of well-being. *Social Science Research*, *35*(4), 1103–1119.

Salvo, L., & Castro, A. (2013). Confiabilidad y validez de la escala de impulsividad de Barratt (BIS-11) en adolescentes. *Revista Chilena de Neuro-Psiquiatría*, *51*(4), 245–254.

Torlay, L., Perrone-Bertolotti, M., Thomas, E., & Baciu, M. (2017). Machine learning-XGBoost analysis of language networks to classify patients with epilepsy. *Brain Informatics*, *4*(3), 159–169. https://doi.org/10.1007/s40708-017-0065-7

Wechsler, D. (2003). Escala Wechsler de Inteligencia para adultos-III. *Mexico City, Mexico: Manual Moderno*.

Wells. (2010). Resilience in Older Adults Living in Rural, Suburban, and Urban Areas. *Online Journal of Rural Nursing and Health Care*. https://doi.org/10.14574/ojrnhc.v10i2.55

Wells, A., & Cartwright-Hatton, S. (2004). A short form of the metacognitions questionnaire: properties of the MCQ-30. *Behaviour Research and Therapy*, *42*(4), 385–396.

Zeng, X., & Luo, G. (2017). Progressive sampling-based Bayesian optimization for efficient and automatic machine learning model selection. *Health Information Science and Systems*, *5*(1), 2. https://doi.org/10.1007/s13755-017-0023-z

Zheng, H., Yuan, J., & Chen, L. (2017). Short-Term Load Forecasting Using EMD-LSTM Neural Networks with a Xgboost Algorithm for Feature Importance Evaluation. *Energies* , Vol. 10. https://doi.org/10.3390/en10081168
